# Thyroid Metastasis of Gastric Cancer: A Rare Occasion With Poor Prognosis

**DOI:** 10.4021/gr230w

**Published:** 2010-09-20

**Authors:** Tomofumi Miura, Junichiro Nakamura, Keita Kimura, Satoshi Yamada, Tsutomu Miura, Masahiko Yanagi, Hajime Yamazaki, Hiroyuki Usuda, Iwao Emura, Toru Takahashi

**Affiliations:** a Division of Gastroenterology and Hepatology, Nagaoka Red Cross Hospital, Japan; b Division of Diabetes and Endocrinology, Nagaoka Red Cross Hospital, Japan; c Division of Nephrology, Nagaoka Red Cross Hospital, Japan; d Division of Medical Technology, Nagaoka Red Cross Hospital, Japan; e Division of Pathology, Nagaoka Red Cross Hospital, Japan

**Keywords:** Gastric cancer, Thyroid metastasis, Fine needle aspiration biopsy

## Abstract

A 68-year-old man was diagnosed as having advanced gastric cancer. Computed tomography showed a thyroid tumor with trachea deviation. This tumor exhibited mosaic echogenecity in ultrasonography. Signet-ring cell carcinoma was found by means of fine needle aspiration biopsy. This tumor gradually became swollen and the thyroid hormone levels in blood were increased without any clinical symptom. Shortly, he died from his illness in the 29th hospital day. Autopsy disclosed that the left lobe of the thyroid gland was highly invaded by malignant cells and that lymphogenic rather than angiogenic metastasis was highly probable. Thyroid metastasis of gastric cancer is extremely rare. The prognosis is very poor. Ultrasonography is a very useful modality especially when coupled with recently developed fine needle aspiration biopsy in differential diagnosis of thyroid tumors once malignancy is suspected. Therapeutic strategy largely depends on the nature of primary malignant tumor. If the tumor is slowly progressive such as renal cell carcinoma and breast cancer, extirpation of thyroid tumors may extend life expectancy. In conclusion, the metastatic thyroid tumor of gastric cancer is rare and shows poor prognosis. Fine needle aspiration biopsy under ultrasonography is strongly recommended as a useful diagnostic tool.

## Introduction

Thyroid metastasis of gastric cancer is rare and shows poor prognosis [1, 2]. Recently fine needle aspiration biopsy (FNAB) under ultrasonography has been developed to diagnose thyroid tumor. We herein report a case in which FNAB was useful to diagnose thyroid metastasis of advanced gastric cancer.

## Case Report

A 68-year-old man was admitted to our hospital for nausea, vomiting and swallowing difficulty. He was under hemodialysis for chronic renal failure due to diabetic nephropathy for eight years. The cervical, supraclavicular and inguinal lymph nodes were swollen but thyroid gland was not physically palpable.

**Figure 1:**
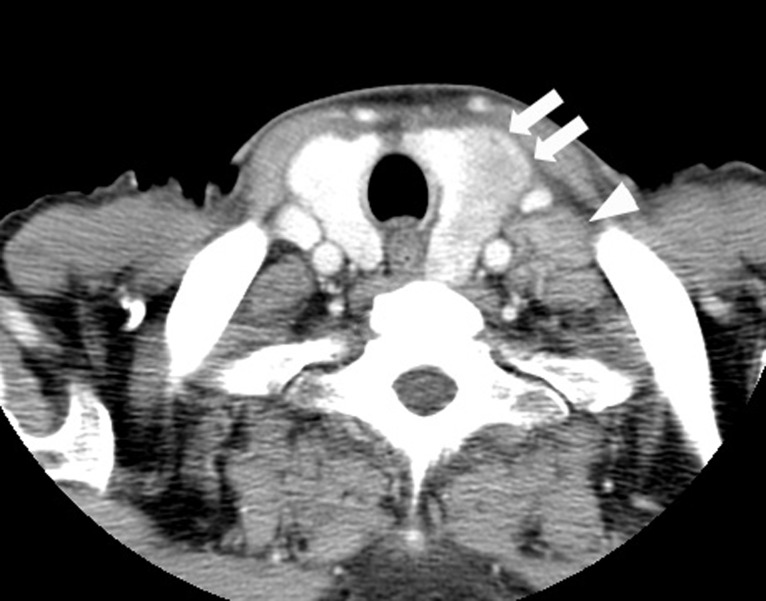
CT findings of the thyroid tumor. Thyroid tumor in the left lobe (arrows) and the left cervical lymph node swelling (arrowhead) are detected in contrast-enhanced CT. This thyroid tumor is a heterogeneously low-density tumor with an unclear border. The trachea is deviated to the right by this tumor.

**Figure 2:**
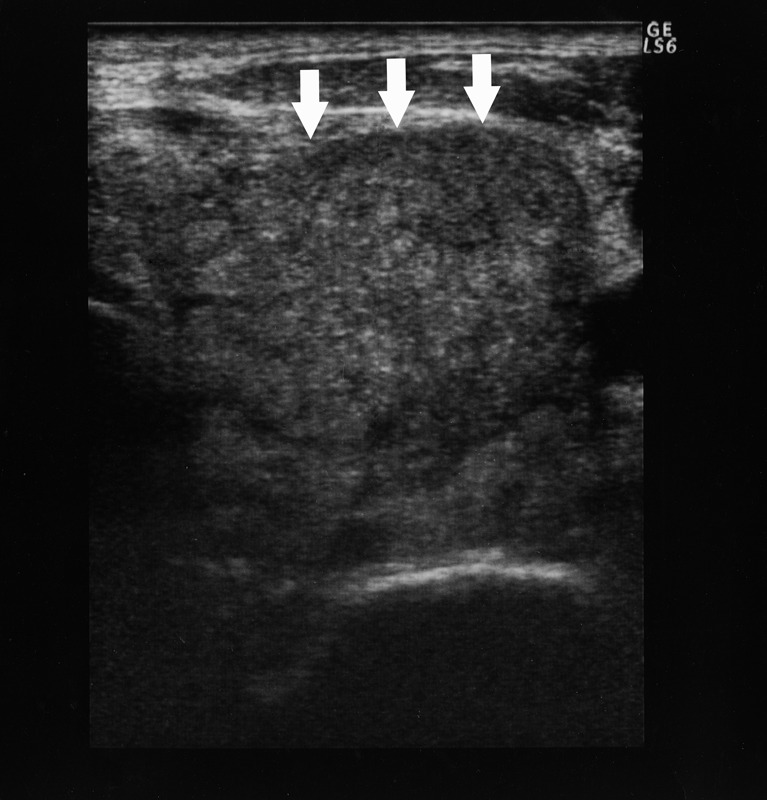
Ultrasonographic findings of the thyroid tumor. Ultrasonography reveals a 3.1 cm-sized tumor in the left lobe of the thyroid gland (arrows). It shows mosaic echogenecity and no calcification inside with a partially unclear border but no apparent spicular formation.

**Figure 3:**
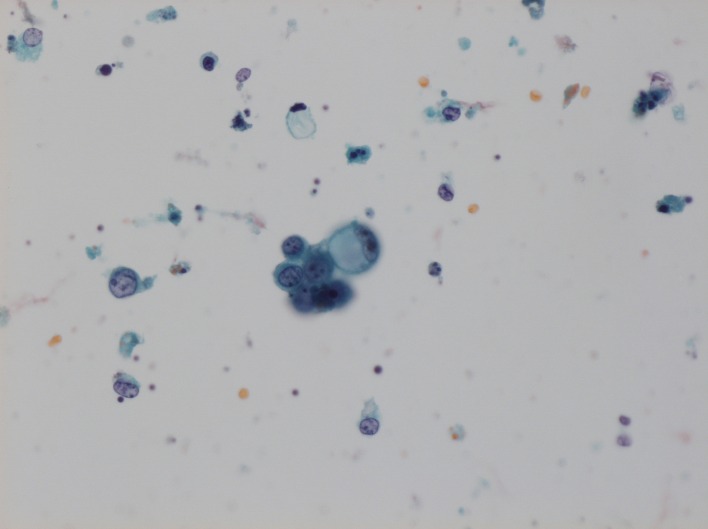
Cytology of the specimen that was obtained by FNAB. A signet-ring cell carcinoma cell and poorly differentiated adenocarcinoma cells with high degree of cellular atypia and a high nucleus/cytoplasm ratio are found by cytology (Papanicolau stain; x 200).

Esophagogastroduodenoscopy revealed advanced gastric cancer with signet-ring cell carcinoma at the upper body of the stomach. It directly invaded to the lower esophagus causing stenosis. Computed tomography (CT) showed generalized lymphadenitis. A low density nodule in the left lobe of the thyroid gland was also found in CT with deviation of trachea (Fig. 1). The thyroid hormone levels in blood were as follows: the free triiodothyronine (fT3) level: 3.27 pg/ml (normal range 2.30 - 4.30 pg/ml); the free thyroxine (fT4) level: 1.35 ng/dl (normal range 0.80 - 1.71 ng/dl); the serum thyrotropin (TSH) level: 0.47 uIU/ml (normal range 0.4 - 4.8 µU/ml). Ultrasonography showed 3.1 cm-sized partially undemarcated tumor with no calcification but with mosaic echogenecity (Fig. 2). Fine needle aspiration biopsy (FNAB) revealed signet-ring cell carcinoma (Fig. 3). Thus he was diagnosed as unresectable advanced gastric cancer with thyroid metastasis. Any radical therapy including surgery or chemotherapy was out of consideration other than palliative care because of his severe illness.

The left lobe of the thyroid gland was gradually enlarged without clinical symptoms such as dyspnea and wheezing. The blood level of thyroid hormones was increased as bellows: that of fT3 was 13.5 pg/ml and fT4 was 2.04 ng/dl at the 17th hospital day; that of fT3 was 17.56 pg/ml and fT4 was 2.56 ng/dl at the 22th hospital day although there were no symptoms of hyperthyroidism. This elevated thyroid hormone level was considered to be the result of the secondary hyperthyroidism due to the tissue destruction by the metastatic thyroid tumor. His illness progressed and finally he died from his illness at the 29th hospital day.

**Figure 4:**
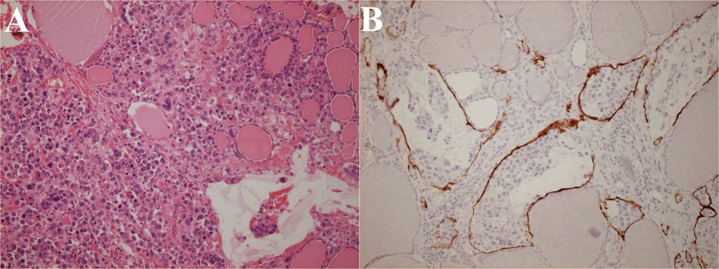
Histopathology of the thyroid gland at autopsy. The thyroid gland is destructed by extensive tumor invasion and colloid content is diminished (A: Hematoxylin and eosin stain; × 200). Lymph vessels are filled with tumor cells (B: Immunohistochemical stain using D2-40 monoclonal antibodies that specifically stain lymph vessels; × 200).

The autopsy results were as follows: Bormann type 3 gastric cancer at the lesser curvature of the upper body was found with direct invasion to the omentum minus and to the hepatic hilum. This tumor was histologically composed of a mixture of signet-ring cell carcinoma and poorly differentiated adenocarcinoma. The thyroid gland was aggressively invaded by tumor cells and colloid was diminished by tissue destruction and the lymph vessels were filled with tumor cells (Fig. 4A, 4B). The nearly same findings were found in other organs. It was concluded based on these findings that he died from highly progressive gastric cancer with generalized metastasis to the whole body most probably through the lymphatic pathway.

## Discussion

The thyroid grand is relatively a rare target organ of metastasis. The frequency of metastatic thyroid tumors in the extirpated thyroid tumors ranges from 0.05% in Mayo Clinic [1] to 0.3% in Ito Hospital [2]. In autopsy cases, it ranges from 1.2% to 24% [3, 4]. The major primary sites of metastatic thyroid tumor are renal cell carcinoma, lung cancer and breast cancer [3, 5]. As to the thyroid metastasis from the digestive tracts, gastric cancer (3.9%) is much less likely than esophageal cancer (18.8%) and colorectal cancer (8.9%) in the Japanese autopsy series [6]. The reason why thyroid gland is uncommon site of metastasis is as follows: 1) rapid and abundant blood supply inhibits tumor implantation, called as “flushing” action; 2) high oxygenation and colloid with rich iodine inhibit tumor progression since malignant cells rather prefer anaerobic metabolism [7].

Metastatic thyroid tumor mimics primary thyroid tumor in terms of imaging characteristics and pathology [8]. Papillary and follicular tumors that are common histology in primary thyroid tumors are especially difficult to distinguish from secondary tumors from other organs [8]. The ultrasonography (US) is now widespread and non-invasive, and shows high sensitivity with low specificity in the detection of thyroid tumors [8]. The US findings such as a taller-than-wide shape, a speculated margin, marked hypoechogenicity, microcalcification and macrocalcification, predict malignancy of thyroid tumors in Korean multicentre retrospective study [9]. Fine needle aspiration biopsy (FNAB) is one of the best ways to diagnose metastatic thyroid tumors with 80-90% sensitivity [3, 4, 8, 10, 11].

Thyroid metastasis is often a part of generalized metastasis to other organs. Thus, prognosis is poor and the averaged residual mean life span is approximately three months [4, 12]. There is a question whether the resection of metastatic thyroid tumor could elongate survival. In the case of rapid progressing cancer such as lung and gastric cancer, life expectancy cannot be extended but in the case of slowly progressing one such as renal cell carcinoma and breast cancer it can be elongated in the previous literatures [4, 10, 12]. Thus, radical treatment is recommended in the latter cases.

There have been only three reported cases in which gastric cancer became metastatic to thyroid gland [10, 11, 13]. In two cases [10, 11] metastasis was hematogeneous and in the other it was lymphogeneous [10, 11, 13]. Hematogenous metastasis from gastric cancer to thyroid gland could occur through the vertebral vein plexus where tumor cells could be spread without passing the lung capillaries [2, 5, 14]. In our case, generalized lymph node swelling was seen and lymph ducts in thyroid gland and in other organs were filled with tumor cells. Hence lymphogeneous metastasis is highly suspected. Previously reported all three cases had symptoms where diagnosis was made by FNAB in one, by open biopsy in another and by thyroidectomy in the last one. Thyroid hormone levels were under upper normal limits in all cases. Prognosis was poor spanning one to six months.

We conclude that thyroid metastasis of gastric cancer is extremely rare and usually shows poor prognosis. FNAB is useful in diagnosis. We should always be aware of the possibility of metastasis from malignancy in other organs whenever we see thyroid tumors.
